# Economic Policy Uncertainty and Financial Innovation: Is There Any Affiliation?

**DOI:** 10.3389/fpsyg.2021.631834

**Published:** 2021-05-24

**Authors:** Zeng Jia, Ahmed Muneeb Mehta, Md. Qamruzzaman, Majid Ali

**Affiliations:** ^1^School of International Programs, Guangdong University of Finance, Guangzhou, China; ^2^Hailey College of Banking and Finance, University of the Punjab, Lahore, Pakistan; ^3^School of Business and Economics, United International University, Dhaka, Bangladesh; ^4^Hailey College of Commerce, University of the Punjab, Lahore, Pakistan

**Keywords:** financial innovation, economic policy uncertainty, ARDL, nonlinear ARDL, Toda-Yamamoto causality test, BRIC, G23, D04, D81

## Abstract

The impetus of this study is to gauge the nexus between economic policy uncertainty (EPU) and financial innovation in Brazil, Russia, India, China, and South Africa (BRIC) nations for the period from 2004M1 to 2018M12. This study utilizes both the linear and non-linear autoregressive distributed lag (ARDL) models to evaluate the long-run and the short-run association between EPU and financial innovation; furthermore, the causal effects are investigated by following the non-Granger casualty framework. The results of long-run cointegration, i.e., the test statistics of modified *F*-test (FPSS), standard Wald test (WPSS), and tBDM, reject the null hypothesis and establish the presence of the long-run association between EPU and financial innovation. Conversely, long-run asymmetry cointegration revealed the test statistics of FPSS, WPSS, and tBDM in non-linear estimation. Furthermore, both in the long run and short run, the Wald test results disclose asymmetric effects running from EPU to financial innovation. In regards to the asymmetric impact of EPU on financial innovation, this study documents that the positive and negative shocks in EPU are negatively linked with financial innovation in the long run but are insignificant for short-run effects. Besides, financial innovation measured by R&D investment exhibits a positive linkage with shocks in EPU, implying that uncertainty induces innovation in the economy. Referring to causality effects, this study divulges the *feedback hypothesis*, i.e., bidirectional causality prevails between EPU and financial innovation in all sample countries.

## Highlights

- A first-ever empirical study for investigating economic policy uncertainty and financial innovation.
- Financial innovation measured using three proxies in empirical models.
- Both symmetric and asymmetric effects investigated applying ARDL and NARDL.
- NARDL revealed asymmetry both in the long-run and short-run.
- Feedback hypothesis holds for explaining causality between EPU and financial innovation [EPU←→ FI].

## Introduction

This study hypothesizes that economic policy uncertainty, hereafter EPU, influences financial innovation; however, their association is yet to be explored in the empirical literature. Therefore, it is necessary to scrutinize the proposed hypothesis due to the significance and critical role of financial innovation in the economy, especially in the financial sector. Finance scholars, including Miller (1986) and Merton (1992), advocated that innovative financial products and services play a critical role in increasing the institutional efficiency and sustainability, thus eventually accelerating economic progress. Furthermore, financial innovation expands existing financing opportunities by lowering the cost of funds and efficient financial intermediation.

Since the seminal work of Schumpeter (1911), the term “financial innovation” has attracted an audience. Since then, researchers and academicians have been investing considerable time in gauging the effects of financial innovation on various participants in the economy. The empirical literature produces two lines of thoughts regarding the role of financial innovation in the economy. A group of researchers exposed the positive impact of financial innovation on economic growth (Michalopoulos et al., 2009; Ajide, 2015; Laeven et al., 2015; Bara and Mudxingiri, 2016; Bara et al., 2016; Qamruzzaman and Jianguo, 2017; Qamruzzaman and Wei, 2019), financial sector development, (Malak, 2013; Otoo, 2013; Domehe et al., 2014), foreign direct investment (Qamruzzaman and Jianguo, 2018a), and financial inclusion (Qamruzzaman and Wei, 2019). The second vein of thought in the empirical literature, i.e., harmful or advise effects based on firm-specific and country-specific investigation (for instance, Smith et al., 1990) pointed to increased volatility. However, the positive impact of financial innovation is more prominent than a negative one. Tufano (2003) instituted that financial innovations are crucial for global financial integration and diversification; it is because innovation in financial products and services enables investment diversification and risk mitigation. That is why it is rightly said that financial innovation is a tool for investment risk mitigation through diversification.

Evolvement and diffusion of financial innovation persistently seek a pleasant environment, especially in the financial system. Therefore, with this study, we intended to explore new evidence regarding dose EPU influences on financial innovation, if yes, then in which direction? Recently, EPU has received attention from researchers and academicians; in this process, a vast number of studies appear in the literature dealing with the impacts of EPU on a financial system, such as credit expansion (Nodari, 2014; Bordo et al., 2016; Chi and Li, 2017; Nguyen et al., 2020), financial stability (Li and Zhong, 2020; Phan et al., 2020), and banking activities (Chi and Li, 2017; Lee et al., 2017; Tran et al., 2020). The literature suggests that the key players of the financial system are vulnerable due to the changes in the present state of EPU in the economy. Furthermore, empirical literature also revealed a diverse outcome dealing with EPU impact on stock market volatility (Liu and Zhang, 2015), the stock price (Ko and Lee, 2015; Phan et al., 2018), financial market (Karnizova and Li, 2014; Arouri et al., 2016; Chen et al., 2017; Tsai, 2017), exchange rate volatility (Krol, 2014), firm-level investment (Kang et al., 2014; Wang et al., 2014), unemployment (Caggiano et al., 2017), stock return (Li et al., 2016; You et al., 2017), capital structure (Zhang et al., 2015), and so on. The possible indirect relationship between EPU and financial innovation can be detected with careful consideration of variables. EPU influences those because EPU and financial innovation appear as the key determinants for several macrovariables in the literature.

The *novelty* of this study relies on the following aspects. First, to our best knowledge, this is the first-ever empirical study to investigate the nexus between financial innovation and EPU in BRIC nations for the period 2004M1–2018M12. The underlying motivation for the selection of BRIC nations is data availability, especially the data related to EPU; in addition, according to the existing literature, the selected nations possess some common dynamics, such as bilateral trade association, technological expertise, and economic activities (Tseng, 2009; Marr and Reynard, 2010; Arif et al., 2020). Therefore, the selection of BRIC nations in the empirical investigation can have a potential comparative assessment, and we followed the prevailing thoughts. Second, measurement of financial innovation in the empirical literature is one of the critical issues because of conclusive consensus yet to be established for proxy; furthermore, the empirical findings with a single proxy measure for financial innovation may not produce such enthralling results, and this study considers three widely used proxies for measuring the presence of financial innovation in the empirical equation. Third, to gauge the possible association between EPU and financial innovation, this study applies the non-linear unit root test proposed by Kapetanios et al. (2003) and Kruse (2011), autoregressive distributed lag (ARDL) model initiated by Pesaran et al. (2001), and non-linear ARDL proposed by Shin et al. (2014). Also their possible directional causality was investigated by performing the non-Granger causality test proposed by Toda and Yamamoto (1995).

The remaining sections apart from the introduction are the “Literature review” section, dealing with the literature survey. The definitions of variables and econometrical methodology are described in detail in the “Data and methodology of this study” section. The empirical model estimation and interpretation are discussed in the “Model estimation and interpretation” section. Finally, the study findings and the reports of policy implications are described in the “Findings and policy implications” section.

## Literature Review

After the financial crisis of 2008, the world economy feels the importance of effective and stable economic policy to regain financial soundness. It is because economic stability accelerates sustainable economic growth by lessening the adverse shocks in macro fundamentals. Conversely, global economic integration and macro complexity produce economic uncertainty and adverse shocks in economic activities both in the long run and the short run. The studies by Krol (2014) are intertwined, and the complex nature of macro fundamentals in the market economy plays a positive role toward economic uncertainty. Economic uncertainty, according to Baker and Martin (2011), shakes economic activities both at the macro and micro levels and reduces confidence in the economy from the perspective of domestic and foreign investors. Besides, from an investment viewpoint, firms avail benefits from uncertainty by delaying the investment on the ground of higher cost and costly workforce to run the project (Bernanke, 1983).

### Effects of Financial Innovation

Capital accumulation, reallocation, and economic resource mobilization play a critical role in achieving economic sustainability in the presence of a well-functioning financial sector. Regulatory bodies persistently seek to formulate and implement effective monetary and fiscal policies to ensure financial efficiency. In particular, financial efficiency demands diversification in terms of financial services and products so that a larger population can serve with ease; in a study, Miller (1986) advocated that innovativeness in the financial sector intensifies the growth of a financial sector by offering versatile investment opportunities and risk diversification mechanisms, in particular financial markets. He also postulated that diversified financial assets assist in transferring risk, maximizing investment returns from tax-deductible security, and financing projects with accumulating marginal investors.

In the modern economy, the effects of financial innovation, i.e., both positive and negative, are appreciated and acknowledged in the empirical literature, especially in the field of the financial system. Regarding the positive impact of financial innovation, the world economy has been observing global financial integration and the expansion of existing financial assets and services. By adapting and diffusing innovative products and services in the financial system, financial institutions have a greater scope to serve a large group of the population, especially those who are not enlisted in the formal financial system. Financial innovation works for financial inclusion by offering risk-diversified financial products and services in the financial system. Besides, financial innovation drives financial progress through capital adequacy, investment opportunity, and financial intermediation with fetching efficiency in the capital market. Apart from bank-based financial institutions' development, financial innovation also plays a pivotal role in developing non-bank financial institutions such as leasing companies and insurance companies. The developing economies in financial regions incorporate commercial banks, leasing institutions, insurance companies, and specified financial institutions, such as financial markets, informal financial companies, and house building finance corporations.

Financial literature, especially financial growth, postulates that financial innovation contributes to overall economic growth by offering unique prospects in the financial system, including efficient financial intermediation, financial diversification, economic resources reallocation, and financial inclusion. The role of financial innovation assessed in the empirical literature focuses on diversified macro fundamentals, such as increasing the value of financial products and facilities (McGuire and Conroy, 2013), raising capital growth and distribution practices (Allen, 2012; Uddin et al., 2014), advancing the practices of financial development (Ozcan, 2008), and upsurging the efficacy of financial institutions (Shaughnessy, 2015).

Over the past decades, financial innovation has contributed to the enormous evolvement in the hunt for financial inclusion. Possibly, the most prominent example of this is the accomplishment of mobile money transfer and banking services. In this vein, a growing number of studies are available in the empirical literature. For instance, in the study by Qamruzzaman and Wei (2019), they advocated that the process of financial inclusion has been augmented by the diffusion of innovative financial products and services in the economy. Furthermore, Arslanian and Fischer (2019) suggested that financial innovation, particularly technological advancement in providing financial services, results in easy access to the unbanked population in the formal financial system. Similar findings are available in the literature (Agoba et al., 2017; Amoah et al., 2020; see, for instance, Niankara and Muqattash, 2020). So it is possible to believe that financial innovation broke the chain of demographic and social attribute issues that are dragging people to avail financial benefits.

According to a study by Dunne and Kasekende (2018), the money demand in sub-Saharan Africa is adversely influenced by financial innovation both in the long run and short run. They suggested that financial innovation induces people to move from liquidating currency to electronic currency in their daily transactions, and thus the state of money supply falls, leading to disruption. Further evidence is available in the studies by Dooley and Spinelli (1989), Arrau and De Gregorio (1993), Arrau et al. (1995), Hafer and Kutan (2003), Adil et al. (2020), and Dlamini and Mabuza (2020). Existing literature advocates that financial innovation not only plays a critical role in money demand functions but also assists in achieving transactional efficiency in the financial sector as a result of the adaption and evolvement of innovative financial services in the financial system. Malik and Aslam (2010) postulated that financial innovation brings changes in the financial sector through augmenting the necessity of banking industry reformation, strict policy formulation and implementation, and stability in the financial transaction.

Another vein includes financial innovation and financial stability. Financial innovation is the act of creating and popularizing new financial instruments and new financial technologies, institutions, and markets. Xin (2009) advocated that financial assets innovation demands effective regulatory establishment to mitigate financial risk because diversifications can act as a double-edged sword in the financial system. However, risk diversification with efficiency is one of the benefits of financial innovation, which plays a critical role in establishing financial stability. Lüke and Gaowang (2014) revealed the financial stability of several variables, including financial assets price in the financial market, economic uncertainty, economic shocks, and behavior of bank-based financial institutions. They also detected that financial market capacity, preference of an investor, and financial assets performance immensely rely on financial stability.

## Effects of Economic Policy Uncertainty

Economic policy uncertainty, over the past decades, has emerged as a critical determinant in the economy, regardless of the state of the economy. To explore the true impact of EPU, a growing number of researchers have invested considerable time gauging the nexus between EPU and the macro and micro fundamentals of the economy. For instance, EPU has an impact on economic growth (Balcilar et al., 2020; Xu et al., 2021), stock market volatility (Liu and Zhang, 2015), stock price (Ko and Lee, 2015; Phan et al., 2018), financial market (Karnizova and Li, 2014; Arouri et al., 2016; Chen et al., 2017; Tsai, 2017), exchange rate volatility (Krol, 2014), firm-level investment (Kang et al., 2014; Wang et al., 2014), unemployment (Caggiano et al., 2017), stock return (Li et al., 2016; You et al., 2017), and capital structure (Zhang et al., 2015). Another set of findings was also available in the empirical literature: macro factor effects on EPU, for instance, oil price shocks (Antonakakis et al., 2014), gold, and Bitcoin (Wu et al., 2019).

In a study, Nguyen et al. (2020) revealed the adverse effects of EPU on credit growth in both advanced and developing nations. However, the magnitude of the coefficients exposes that emerging economies are more vulnerable than advanced economies. In another study, Phan et al. (2020) postulated that EPU is the key determinant for causing financial stability in the economy. They also suggested that the impact of EPU on financial stability is stronger for countries with higher competition, lower regulatory capital, and smaller financial systems. Chi and Li (2017) observed that EPU plays a positive role in increasing loan defaulters in the financial institutions in China and thus forced financial institutions to decrease the loan size. Panousi and Papanikolaou (2012) documented that high EPU can increase financing costs and risk aversion among top managers, which depresses the investment size. Besides, the depressing effect of EPU on investments is more significant in firms with higher irreversibility in investment which are more dependent on government public expenditure (Gulen and Ion, 2016).

A study was performed by Uzuner et al. (2020) for detecting the association between tourist arrival and EPU with panel data consisting of France, Germany, the United Kingdom, and the United States for the period 1985Q1–2017Q4 with a causality test. The study findings reveal that migrants affect decision-making about EPU, and along with policy instability, they could also have an effect on economic development. Moreover, the study suggests that migration had a huge impact on the market success of the four main destinations of the world for foreign tourism. Another study has been performed by Akadiri et al. (2020) to evaluate the possible association between EPU and tourism with heterogeneous panel data with a causality test for the period spanning 1995–2016. The study findings disclose that the feedback hypothesis holds for explaining the causal effects between EPU and tourism in France, Ireland, and the United States and unidirectional causality running from tourist arrivals to EPU in Brazil, Canada, China, and Germany. The impact of EPU on the housing market and agricultural land was investigated by Alola and Uzuner (2020) with a panel of 15 counties by employing panel cointegration. The study findings suggest that the *feedback hypothesis* holds for explaining the causality between agricultural lands and the housing market.

No conclusive pronouncement is available in the existing literature regarding the nexus between EPU and financial innovation. Considering, however, their impact on macro and micro fundamentals, it is apparent that both variables play a deterministic role but in diverse directions. Financial innovation augments financial development offering versatile financial products and services to the economy, especially for unbanked pollution. It is suggested that financial inclusion is one of the results that can be observed in the economy. On the other hand, EPU induces financial instability with fragile financial systems, discouraging people from becoming involved in the formal financial system. In a study by Li and Zhong (2020), it was asserted that EPU shocks are adversely linked with financial market volatility. The study documents that EPU increases financial volatility through interest rate movement, exchange rate fluctuation, stock price declination, and housing price reduction in the financial system.

Furthermore, the nexus of financial innovation that leads to financial volatility exposes negative associations, i.e., risk diversification is one of the benefits of adopting innovation in the financial sector. However, Xin (2009) documented that excessive financial innovation is a curse for the financial sector. Furthermore, Li and Zhang (2010) revealed that investor irrational behavior causes financial instability in the long run.

Considering the indirect approach to establish the interlink between financial innovation and EPU, one common verdict can be observed in the financial sector, i.e., rules and regulations about the financial system influence both. Hence, we can presume that there may be an empirical association available between financial innovation and EPU.

## Data and Methodology of This Study

This study utilizes monthly time series data for the period from 2004M1 to 2018M12 of BRIC countries. The selection of countries and study period purely relies on data availability. All the variables were extracted from interfacial financial statistics (IFS) published by IMF except the index of EPU.

### Financial Innovation

Lewis and Mizen (2000) posited that innovation in the financial system appeared in either form, i.e., product innovation or process innovation. Product innovation entails advancement in financial assets through modification or adaption of improved financial assets, such as mutual funds, sweep accounts, and pension funds. Process innovation postulates improvement in fund accumulation and reallocation processes, such as automated teller machines, point-of-sale terminals, and electronic funds transfer.

There is no consensus proxy available in the empirical literature because to measure financial innovation in the empirical studies, researchers utilize several proxy variables. Such variations rely on data availability and the way of estimation along with the socioeconomic status of the countries. However, in order to be consistent with the prevailing literature, this study considers three proxy measures that are widely used in various empirical studies. The first proxy is the broad-to-narrow money (M2/M1), which affects the demand for real cash balances, the income, and interest elasticity for money demand (Arrau and De Gregorio, 1993; Ansong et al., 2011; Bara and Mudxingiri, 2016; Bara et al., 2016; Qamruzzaman and Jianguo, 2017, 2018a,c; Nazir et al., 2018; Qamruzzaman and Wei, 2018). For the second measure of financial innovations (FI), this study employs the ratio of M3/M1, following Dunne and Kasekende (2018), Mannah-Blankson and Belnye (2004), Ajide (2015), and Kasekende and Nikolaidou (2014). Furthermore, following the empirical literature, including Bernier and Plouffe (2019), Beck et al. (2016), and Ajide (2015), study considered financial sector R&D expenditures as a percentage of GDP as an another measures for financial innovation.

### Economic Policy Uncertainty

Baker et al. (2016) measured EPU for major countries and regions globally, and the data can be obtained from the EPU database^1^. The database includes uncertainties regarding tax, spending, monetary, and regulatory policy by the government that is calculated from four components, i.e., the frequency of economic policies appeared in the newspaper, the number of expired code, the extent of forecaster disagreement over future inflation, and government purchases.

For control variables, by following the empirical studies dealing with assessing financial innovation effects (see, for instance, Dunne and Kasekende, 2018), this study considers three control variables, namely, GDP growth rate, gross savings as percentage of GDP, and non-performing loans. All data are transformed by taking natural logarithms to correct for potential heteroskedasticity. Descriptive statistics and pairwise correlation are mentioned in Table 1.

**Table 1:** Results of unit root test.

	**Brazil**		**Russia**		**India**		**China**	
**Panel A: unit root test with ADF test with Constant and Constant & Trend**								
F1	−1.195	−1.634	−1.553	−4.918a	−1.6843	−2.555	−1.054	−1.331
F2	1.060	−2.115	−1.797	−4.321a	−0.9434	−3.036	−1.356	−2.003
F3	−1.109	−2.081	−2.718c	−2.681c	−1.9415	−1.867	−0.778	−3.514
PE	−5.521a	−6.807a	−0.569	−1.418	−5.3406c	−10.537a	−0.778	−3.514
GS	0.378	−1.61	−1.702	−1.910	−0.6817	−0.7999	−1.660	−2.143
BL	−6.429a	−6.006a	−1.558	−1.357	−2.2964	−2.7459	−0.954	−1.425
Y	−3.158b	−3.142c	−1.015	−1.911	−1.5256	−1.1931	−1.092	−1.789
ΔF1	−13.369a	−13.369a	−9.901a	−9.888a	−2.6452c	−2.9395	−16.738a	−16.696a
ΔF2	−16.570a	−16.770a	−9.381a	−9.366a	−2.8235c	−2.8185	−16.583a	−16.541a
ΔF3	−26.155a	−25.993a	−16.226a	−16.199a	−4.9243a	−4.9323a	−16.650a	−16.721a
ΔPE	−9.383a	−9.611a	−8.607a	−11.55a	−12.714a	−12.680a	−16.653a	−16.721a
ΔGS	−9.388a	−9.378a	−3.947a	−3.958a	−4.875	−4.773	−7.255a	−7.322a
ΔBL	−7.377a	−7.642a	−11.241a	−11.251a	−12.879a	−12.862a	−12.930a	−12.896a
ΔY	−12.394a	−12.466a	−13.158a	−13.121a	−12.363a	−12.393a	−13.375a	−13.34a
**Panel A: unit root test with P–P test**								
F1	−1.115	−0.789	−1.8059	−3.7685b	−1.589	−3.905	−1.027	−1.761
F2	1.228	−1.943	−1.4592	−3.4191c	−0.753	−2.70	−1.062	−1.790
F3	−0.804	−2.318	−5.4896a	−5.4781a	−2.756	−2.636	−1.806	−3.107
EPU	−5.536a	−6.673a	−0.657	−1.1768	−8.679a	−10.63a	−1.806	−3.100
GS	0.359	−1.722	−1.6101	−1.7191	−2.140	−2.126	−1.411	−1.836
BL	−2.553	−2.538	−1.8614	−1.8949	−2.534	−2.989	−0.988	−1.536
Y	−3.234b	−3.154c	−1.0301	−1.9809	−1.589	−1.446	−1.092	−1.810a
ΔF1	−12.911a	−12.94a	−19.299a	−20.822a	−21.348a	−23.154a	−16.342a	−16.309a
ΔF2	−17.054a	−17.273a	−14.616a	−14.851a	−19.108a	−19.061a	−16.226a	−16.191a
ΔF3	−14.049a	−14.011a	−29.039a	−29.288a	−9.857a	−9.899a	−17.323a	−17.557a
ΔEPU	−5.455a	−5.711a	−6.802a	−6.796a	−49.777	−49.603	−17.320a	−17.537a
ΔGS	−3.937a	−3.892a	−6.277a	−6.253a	−7.975a	−8.366a	−7.760a	−7.782a
ΔBL	−5.196a	−5.149a	−11.648a	−11.645a	−12.879a	−12.862a	−12.933a	−12.899a
ΔY	−8.443a	−8.541a	−13.158a	−13.121a	−12.473a	−12.460a	−13.375a	−13.34a
**Panel A: unit root test with KPSS test**								
F1	1.585a	0.112	1.5383a	0.1895b	1.6741a	0.1467b	1.2791a	0.1507b
F2	1.637a	0.329a	1.5517a	0.1545b	1.6258a	0.1247c	1.2812a	0.156b
F3	1.443a	0.080	1.3027a	0.2936a	1.1605a	0.089	1.1161a	0.1729b
PE	0.840a	0.145c	1.2799a	0.1537b	1.497a	1.0937a	1.1161a	0.1729b
GS	1.260a	0.262a	1.2107a	0.1098b	1.092b	1.0781a	1.0617a	0.1367b
BL	0.393a	0.149b	1.2681a	0.2653a	0.4262	0.1498b	1.3069a	0.2501a
Y	0.947a	0.089	1.3755a	0.1695b	1.1033a	0.2653a	1.4003a	0.1944b
ΔF1	0.136	0.107	0.1902	0.1812b	1.2131a	0.3796a	0.1078	0.1072
ΔF2	0.398c	0.087	0.1543	0.1392	0.0766	0.0773	0.0941	0.0938
ΔF3	0.067	0.050	0.1451	0.108	0.0966	0.0487	0.2648	0.0846
ΔPE	0.242	0.043	0.2018	0.184b	0.0418	0.0416	0.2648	0.0846
ΔGS	0.068	0.053	0.0939	0.0929	0.2689	0.0758	0.1146	0.0524
ΔBL	0.363	0.1641b	0.1036	0.0478	0.0612	0.0298	0.1424	0.1355
ΔY	0.178	0.064	0.0918	0.092	0.1593	0.0679	0.0941	0.0885

### Estimation Techniques

This study performs several econometric techniques for unveiling certain types of information. Investigating variables for the order of integration, this study applies three traditional unit root tests, namely, ADF (Dickey and Fuller, 1979), P-P (Phillips and Perron, 1988), and KPSS (Kwiatkowski et al., 1992), assuming linear stationary process (see Table 1). Furthermore, the studies by Galadima and Aminu (2020) and Qamruzzaman and Karim (2020) advocate non-linear unit root tests, following Kapetanios et al. (2003) and Kruse (2011), for observing variables for the order of integration with the assumption of a non-linear system (see Tables 2, 3). Furthermore, the Brock–Dechert–Scheinkman (BDS) (Broock et al., 1996) non-linearity test and the non-linear ordinary least squares (NOLS) estimation techniques can also be applied for confirming the possible non-linearity between financial innovation and EPU. The elasticities of non-linear effects, i.e., positive and negative shocks of EPU on financial innovation, are evaluated by applying the non-linear ADRL model familiarized by Shin et al. (2014). Finally, the directional causal relationship is investigated by symmetric and asymmetric effects of EPU on financial innovation by following the non-Granger causality framework introduced by Toda and Yamamoto (1995).

**Table 2:** Results of KSS non-linear unit root test.

**Series**		**FI^**1**^**	**FI^**2**^**	**FI^**2**^**	**EPU**	**BL**	**GS**	**Y**
Case 1	Brazil	−4.751a	−0.718	−2.157	−4.323a	−3.006a	−3.013a	−3.134a
	Russia	−2.751	−3.124a	0.126	−1.376	−4.034a	−1.935	−4.561a
	India	−6.277a	−3.112a	−6.726a	−1.141	−1.388	−5.297a	−4.335a
	China	−6.522a	3.246a	−2.898a	−3.378a	−3.043a	−1.008	−1.121
Case 2	Brazil	−2.517*c*	−6.774a	−9.654	−1.642	−4.951a	−4.406a	−3.978a
	Russia	−2.728*c*	−3.373	−7.528	−3.268*c*	−3.171	−4.806a	−2.57
	India	−6.142a	6.849a	−1.672a	−3.408b	−4.873a	−1.818	−1.277
	China	−6.142a	6.214a	−2.638	−1.574	−5.651a	−5.145a	−3.414b
Case 3	Brazil	−4.517a	−6.782a	−9.124a	−2.21	−1.033	−1.29	−1.767
	Russia	−2.013	−3.171b	−9.210*a*	−2.32	−1.781	−4.145a	−4.577a
	India	4.032a	7.363a	−1.890	−4.911a	−5.455a	−4.408a	−1.78
	China	4.032a	7.634a	−6.811a	−3.514b	−4.859a	−1.175	−2.089
**Critical value level Kapetanios et al.**, **2003**								
		**Case 1**	**Case 2**	**Case 3**				
	1%	−2:82	−3:48	−3:93				
	5%	−2:22	−2:93	−3:40				
	10%	−1:92	−2:66	−3:13				

**Table 3:** Results of Kruse non-linear unit root test.

**Series**		**FI^**1**^**	**FI^**2**^**	**FI^**2**^**	**EPU**	**BL**	**GS**	**Y**
Case 1	Brazil	24.943^***^	0.921	11.634^***^	12.066^***^	7.949	4.077	13.266^***^
	Russia	35.526^***^	18.064^***^	10.929^*^	18.654^***^	15.454^***^	12.236^***^	5.51
	India	12.841^***^	14.575^***^	15.115^**^	7.749	5.353	10.927^***^	9.268
	China	9.874^**^	38.126^***^	5.664	17.914^***^	18.391^***^	18.021^***^	6.203
Case 2	Brazil	14.009^***^	13.064^***^	17.198^***^	10.863^**^	10.446^**^	6.328	19.438^***^
	Russia	11.267^***^	16.524^***^	9.383	18.014^***^	17.364^**^	8.665^*^	4.945
	India	5.947	3.280	13.954^**^	3.358	10.091^*^	2.437	8.925^*^
	China	15.748^***^	13.046^***^	6.286	17.126^***^	18.541^***^	9.881^*^	17.102^***^
Case -3	Brazil	16.952^***^	12.243^***^	16.048^**^	11.224^***^	12.775^***^	7.276	3.199
	Russia	30.948^***^	5.748	7.150	14.395^***^	14.125^***^	9.911	19.491^***^
	India	11.287^***^	3.780	3.101	7.881	15.546^***^	19.947^***^	7.685
	China	14.214^***^	11.332^***^	5.807	14.327^***^	8.445	15.025^***^	9.629
**Asymptotic critical values of** ***t*****-statistic**								
		**Case 1**	**Case 2**	**Case 3**				
	1%	13.15	13.75	17.10				
	*5%*	9.53	10.17	12.82				
	*10%*	7.85	8.60	11.10				

*The critical values are obtained from the study by Kruse (2011). A denotes the optimal lag length selected by the SBC. The estimation and tests were conducted using a program code written in “R” produced by Kruse*.

***, **, and * *denote the rejection of a unit root null hypothesis at the 1, 5, and 10% significance level, respectively*.

#### The Kapetanios et al. (2003) Test

The performance of conventional unit root tests is under stress due to the conflict between theoretical prediction and test statistics, i.e., the present form of linear unit root tests is incapable to detect theoretical prediction and fails to establish it (Rose, 1988; Taylor et al., 2001). With the motivation of mitigating dissatisfaction with a conventional unit root test, Kapetanios et al. (2003) familiarized a nonlinear exponential smooth transition autoregressive (ESTAR) globally stationary process.

Therefore, following Kapetanios et al. (2003), Liu and He (2010), Anoruo and Murthy (2014); and Galadima and Aminu (2020), this study specifies the ESTAR model as

(1) $$\begin{array}{l} \Delta Y_{t}=\beta Y_{t-1}\left\{1-\exp\left({-\theta Y}_{t-1}^{2}\right)\right\}+\varepsilon_{t}\;t=1,2\ldots \;T \end{array}$$

where *Y*_*t*_ is the time series of interest, β and θ are unidentified factors, the term $\left\{1-\exp\left({-\theta Y}_{t-1}^{2}\right)\right\}$ specifies the test to characterize the non-linear adjustment, and ε_*t*_ is the stochastic term with a zero mean and a constant variance.

Hence from Equation (1), this study tests the following hypothesis:

(2) $$\begin{array}{l} H_{0}:\;\theta =0 \end{array}$$

and

(3) $$\begin{array}{l} H_{1}:\;\theta >0 \end{array}$$

In addition to the reparameterization of Equation (1), to obtain a first-order Taylor series approximation to the ESTAR model under the null and get the auxiliary regression:

(4) $$\begin{array}{l} \Delta Y_{t}=\delta Y_{t-1}^{3}+\text{error} \end{array}$$

This suggests that it is easy to get the value of *t*-statistics for δ = 0 against δ < 1 as,

(5) $$\begin{array}{l} t_{NL}=\frac{\hat{\delta }}{SE\left(\hat{\delta }\right)} \end{array}$$

where $\hat{\delta }$ is the ordinary least squares (OLS) estimates of *d* and SE$\left(\hat{\delta }\right)$ is the standard error of the ^∧^*d*. Nonetheless, it is noteworthy that the *statistic t*_*NL*_ does not follow an asymptotic standard normal distribution.

#### The Kruse (2011) Test

Kapetanios et al. (2003) proposed an ESTAR-based non-linear unit root test by assuming that the location parameter *c* in the smooth transition function is equal to zero (see Equation 1) for empirical study and became popular among researchers. However, a growing number of studies observe that the coefficient of *c* is significant (e.g., Michael et al., 1997; Sarantis, 1999; Taylor et al., 2001; Rapach and Wohar, 2006). Kruse (2011) argued that the exclusion of basic assumptions leads to the non-standard testing problem. Therefore, a modified version of test statistics was used by Abadir and Distaso (2007) to mitigate the location parameter issue. Eventually, the following modified ESTAR specification appeared:

(6) $$\begin{array}{r} \Delta Y_{t}=\alpha Y_{t-1}+\delta Y_{t-1}\left\{1-\exp{\left(-\theta (Y_{t-1}-c\right)}^{2}\right\}+\varepsilon_{t}\\ t=1,2\ldots \;T \end{array}$$

where ε_*t*_ ~ iid(0, σ^2^). If the smoothness parameter γ approaches 0, the ESTAR model becomes a linear AR(1) model, i.e., *Y*_*t*_ = α*Y*_*t*−1_ + ε_*t*_ that is stationary if −2 < α < 0. Nonlinear OLS and hence the modified ADF regress as follows:

(7) $$\begin{array}{l} \Delta Y_{t}=\overset{p}{\underset{j=1}{\sum }}\alpha_{j}Y_{t-j}+\gamma_{1}Y_{t-1}^{3}+\gamma_{2}Y_{t-1}^{2}+\varepsilon_{t}\;t=1,2\ldots \;T \end{array}$$

In the equation, the null hypothesis *H*_0_ : θ = 0 turns out γ_1_ = γ_2_ = 0 with the alternative hypothesis of γ_1_ < 0; γ_2_ ≠ 0, where γ_2_ is derived from the fact that the location parameter “*c*” is allowed to take non-zero values.

#### Linear ARDL

Conventional cointegration tests possess certain limitations, and therefore the researchers are persistently seeking alternative ways of evaluating the long-run association in empirical studies. Pesaran et al. (2001) familiarized the OLS-based cointegration test with variables in different orders of integration. Additionally, the short-run adjustment speed toward long-run equilibrium also originates using the linear transformation (Banerjee et al., 1993).

A simplified ARDL model (see Paul, 2014) for these variables *X, Y*, and *Z* can be expressed as:

(8) $$\begin{array}{l} \Delta y_{t}=\;\emptyset_{1}+\gamma_{1}y_{t-1}+\;\gamma_{2}x_{t-1}+\gamma_{3}z_{t-1}+\theta_{1}\\ \overset{n}{\underset{i=1}{\sum }}y+\theta_{2}\overset{n}{\underset{i=1}{\sum }}x+\theta_{3}\overset{n}{\underset{i=1}{\sum }}z+\varepsilon_{1t} \end{array}$$

where γ_1_, γ_2_, *and γ*_3_ are long-run coefficients whose sum is equivalent to the error correction term at the vector error correction model (VECM) model and θ_1_, θ_2_, and θ_3_ denote short-run coefficients.

The generalized ADRL model for gauging the nexus between EPU and financial innovation is as follows:

(9) $$\begin{array}{l} {\Delta FI^{1}}_{t}=\;\alpha_{0}+\beta_{i}{FI^{1}}_{t-1}+\beta_{2}\text{EPU}{\;}_{t-1}+\beta_{3}{\text{BL}}_{t-1}+\beta_{4}GS_{t-1}\\ \;+\beta_{5}{\text{Y}}_{t-1}\;+{\overset{m1}{\underset{j=1}{\sum }}\lambda_{0}{\Delta FI^{1}}_{t-j}}_{0}+\overset{m2}{\underset{j=1}{\sum }}\lambda_{1}\Delta EPU_{t-j}\\ \;+\overset{m3}{\underset{j=0}{\sum }}\lambda_{2}{\Delta BL}_{t-j}+\overset{m4}{\underset{j=0}{\sum }}\lambda_{3}{\Delta GS}_{t-j}+\overset{m5}{\underset{j=0}{\sum }}\lambda_{4}{\Delta Y}_{t-j}+\varepsilon_{t} \end{array}$$

where α is an intercept, β_1_, …, β_6_ represent the long-run coefficients of the empirical model, λ_0_, …, λ_5_ denote short-run coefficients, ε_*t*_ denotes the error correction term, and *m*1, *m*2, *m*3, *m*4, *m*5, and *m*6 are the optimal lags for the first difference variables, which are selected by the Akaike Information Criterion (AIC).

To implement the ARDL model, the OLS method is employed to estimate Equation 9, and then cointegration between the variables can be established in three different ways: first, using the *F*-test studied by Pesaran et al. (2001) with the null hypothesis of “no-cointegration” (*H*_0_ = β_1_ = β_2_ = β_3_ = β_4_ = β_5_ = 0) against the alternative hypothesis of cointegration (*H*_0_ = β_1_ ≠ β_2_ ≠ β_3_ ≠ β_4_ ≠ β_5_ ≠ 0); second, performing a standard Wald test (WPSS), which also tests the above joint null hypothesis; and third, the tBDM test statistic studied by Banerjee et al. (1998) with the null hypothesis of no-cointegration (*H*_0_ : β_1_ = 0) against the alternative of cointegration (*H*_0_ : β_1_ < 0). The testing procedure uses two critical bounds: upper and lower. If the values of the FPSS, WPSS, or tBDM statistics exceed the upper bound, the null hypothesis is rejected. If the test statistics lies below the lower critical bound, the null hypothesis cannot be rejected, and if test statistics lies between the critical bounds, the test is inconclusive.

#### Non-linear ARDL

To gauge the asymmetric effects of EPU on financial innovation, this study employs a non-linear framework widely known as NARDL, which was initiated by Shin et al. (2014), and generalizes the following asymmetric long-run regression:

(10) $$\begin{array}{l} FI_{t}=\;\left(\beta^{+}EPU_{1,t}^{+}+\beta^{-}EPU_{1,t}^{-}\right)+\delta_{i}X_{i}+\varepsilon_{t} \end{array}$$

where $\beta^{+},\;\beta^{-},\text{ and }\delta_{i}\;$are associated with long-run pavements. β^+^ and β^−^ specify the effect of positive and negative shocks in EPU on financial innovation, and δ_*i*_ measures the effects of control variables in the equation.

The newly introduced non-linear framework enables us to address both the long-run and short-run magnitudes in the equation. Therefore, a growing number of empirical studies have been extensively employed in their respective studies for gauging nexus in empirical equations (see, e.g., Ali et al., 2018; Qamruzzaman and Jianguo, 2018a,b,c). The positive and negative shocks of EPU are represented in the equation by $E_{1,t}^{+}+E_{1,t}^{-}$, which are derived by using the following equation:

(11) $$\begin{array}{l} \{\begin{array}{c} {POS\left(EPU\right)}_{1,t}=\;\sum_{k=1}^{t}lnEPU_{k}^{+}=\;\sum_{K=1}^{T}MAX\left({\Delta lnEPU}_{k},0\right)\\ {NEG\left(EPU\right)}_{t}=\;\sum_{k=1}^{t}lnEPU_{k}^{-}=\;\sum_{K=1}^{T}MIN\left({\Delta lnEPU}_{k},0\right) \end{array} \end{array}$$

Shin et al. (2014) showed that the linear model (9) can transform into a non-linear ARDL by incorporating EPU decomposition in the following Equation (11):

(12) $$\begin{array}{l} \Delta {\text{FI}}_{t}=\;\partial {\text{U}}_{t-1}+\left(\beta^{+}EPU_{1,t-1}^{+}+\beta^{-}EPU_{1,t-1}^{-}\right)+\beta_{3}inf_{t-1}\\ \;+\beta_{4}{\text{Y}}_{t-1}+\beta_{5}fd_{t-1}\;+{\overset{m-1}{\underset{j=1}{\sum }}\lambda_{j}{\Delta FI}_{t-j}}_{0}+\overset{n-1}{\underset{j=1}{\sum }}\\ \;\left(\pi^{+}EPU_{1,t-1}^{+}+\pi^{-}EPU_{1,t-1}^{-}\right)++\overset{m-1}{\underset{j=0}{\sum }}\lambda_{4}{\Delta fd}_{t-j}\\ \;+\overset{m-1}{\underset{j=0}{\sum }}\lambda_{5}{\Delta y}_{t-j}+\varepsilon_{t} \end{array}$$

Equation (11) can transform in the following manner,

(13) $$\begin{array}{l} \Delta {\text{FI}}_{t}=\;\partial e_{t-1}+{\overset{k-1}{\underset{j=1}{\sum }}\lambda_{j}{\Delta FI}_{t-m}}_{0}+\overset{k-1}{\underset{m=1}{\sum }}\\ \;\left(\pi^{+}EPU_{1,t-1}^{+}+\pi^{-}EPU_{1,t-1}^{-}\right)++\overset{k-1}{\underset{m=0}{\sum }}\lambda_{4}{\Delta fd}_{t-m}\\ \;+\overset{m-1}{\underset{m=0}{\sum }}\lambda_{5}{\Delta y}_{t-m}+\varepsilon_{t} \end{array}$$

where ${e_{t-1}=FI}_{t-1}-\left(\delta^{+}EPU_{1,t-1}^{+}-\delta^{-}EPU_{1,t-1}^{-}\right)-\theta inf_{t-1}-\vartheta Y_{t-1}-\tau fd_{t-1}$ is the non-linear error correction term with${\;\delta }^{+}=\frac{-\beta^{+}}{\partial }$; $\delta^{-}=\frac{-\beta^{-}}{\partial }$; $\theta =\frac{-\beta_{3}}{\partial };\;\vartheta =\frac{{-\beta }_{4}}{\partial };\;\tau =\frac{-\beta_{5}}{\partial }\;$ are the long-run parameters. $\partial =\overset{m}{\underset{j-1}{\sum }}\varphi_{j}-1$, $\lambda_{j}=\overset{m}{\underset{i=j+1}{\sum }}\varphi_{i}$ *for j*= *1…., m*. $\delta^{+}=\overset{p}{\underset{j=0}{\sum }}\delta_{j}^{+};\;\delta^{-}=\overset{q}{\underset{j=0}{\sum }}\delta_{j}^{-}$. The short-run adjustments of positive and negative shocks in EPU can be detected by π^+^; π^−^. To gauge the asymmetric relationship between EPU and financial innovation, the following NARDL Equation (13) is applied:

(14) $$\begin{array}{l} \Delta {\text{FI}}_{t}=\;\alpha +\partial {\text{FI}}_{t-1}+\beta^{+}EPU_{1,t-1}^{+}+\beta^{-}EPU_{1,t-1}^{-}+\beta inf_{t-1}\\ \;+\beta Y_{t-1}+\beta fd_{t-1}\;+{\overset{m1}{\underset{j=1}{\sum }}\lambda_{j}{\Delta FI}_{t-j}}_{0}+\overset{m2}{\underset{j=0}{\sum }}\left(\pi^{+}EPU_{1,t-1}^{+}\right)\\ \;+\overset{m3}{\underset{j=0}{\sum }}\pi^{-}EUP_{1,t-1}^{-}++\overset{m6}{\underset{j=0}{\sum }}\lambda_{4}{\Delta fd}_{t-j}+\overset{m7}{\underset{j=0}{\sum }}\lambda_{5}{\Delta y}_{t-j}+\varepsilon_{t} \end{array}$$

The existence of an asymmetric long-run relationship is evaluated by following the same procedure as the linear ARDL, i.e., FPSS and WPSS statistics under the join null hypothesis of no-cointegration (*H*_0_ = β_1_ = β_2_ = β_3_ = β_4_ = β_5_ = β_6_ = 0) against the alternatives of cointegration (*H*_0_ = β_1_ ≠ β_2_ ≠ β_3_ ≠ β_4_ ≠ β_5_ ≠ β_6_ ≠ 0), and the *t*_BDM_-test statistic studied by Banerjee et al. (1998) involves testing the null hypothesis of no-cointegration (*H*_0_ : β_1_ = 0) against the alternative of cointegration (*H*_0_ : β_1_ < 0). When non-linear cointegration is confirmed, the next step is to assess long-run symmetry, i.e., (β^+^ = β^−^) and short-run (additive) symmetry, i.e., ($\sum_{j=1}^{n-1}\left(\pi^{+}E_{1,t-1}^{+}\right)=\sum_{j=1}^{n-1}\pi^{-}E_{1,t-1}^{-})$ by applying a WPSS.

#### Toda Yamamoto Causality Test

The non-causality test proposed by Toda and Yamamoto (1995) is utilized to gauge the directional association between EPU and financial innovation because traditional casualty tests are based on *F*-statistics in a regression context for determining whether model parameters are jointly zero (a stable VAR model), which is not valid when variables are integrated. To overcome the existing limitations with the traditional causality tests, Toda and Yamamoto (1995) proposed a causality test utilizing the modified Wald test to restrict a VAR(*k*). The causality test performed by Toda and Yamamoto (1995) is based on the idea, i.e., vector autoregressive at level (*P* = *K*+*D*_*max*_) with correct VAR order *K* and *d* extra lag, where *d* represents the maximum order of integration of time series.

According to Zapata and Rambaldi (1997), the non-causality test performed by Toda and Yamamoto possesses certain advantages over the traditional Granger causality test. First, assessing causality with a non-causality test does not require cointegration properties in the system equation. Second, in the integration of mixed order of variables, i.e., either *I*(0) or *I*(1), the MWALD test can investigate the existing causality in the empirical equation.

(15) $$\begin{array}{l} {FI^{1}}_{t}=\alpha_{0}+\overset{k}{\underset{i=1}{\sum }}\beta_{1i}{FI^{1}}_{t-i}+\overset{d_{max}}{\underset{j=k+1}{\sum }}\beta_{2j}{FI^{1}}_{t-j}+\overset{k}{\underset{i=1}{\sum }}\gamma_{1i}EPU_{t-i}\\ \;+\overset{d_{max}}{\underset{j=k+1}{\sum }}\gamma_{1j}EPU_{t-j}+\overset{k}{\underset{i=1}{\sum }}\varphi_{1i}Y_{t-i}+\overset{d_{max}}{\underset{j=k+1}{\sum }}\varphi_{1j}Y_{t-j}\\ \;+\overset{k}{\underset{i=1}{\sum }}\delta_{1i}{BL_{vol}}_{t-i}+\overset{d_{max}}{\underset{j=k+1}{\sum }}\delta_{2j}{BL_{vol}}_{t-j}+\overset{k}{\underset{i=1}{\sum }}\theta_{1i}GS_{t-i}\\ \;+\overset{d_{max}}{\underset{j=k+1}{\sum }}\theta_{2j}GS_{t-j}+\varepsilon_{1t} \end{array}$$

(16) $$\begin{array}{l} {FI^{2}}_{t}=\alpha_{0}+\overset{k}{\underset{i=1}{\sum }}\beta_{1i}{FI^{2}}_{t-i}+\overset{d_{max}}{\underset{j=k+1}{\sum }}\beta_{2j}{FI^{2}}_{t-j}+\overset{k}{\underset{i=1}{\sum }}\gamma_{1i}EPU_{t-i}\\ \;+\overset{d_{max}}{\underset{j=k+1}{\sum }}\gamma_{1j}EPU_{t-j}+\overset{k}{\underset{i=1}{\sum }}\varphi_{1i}Y_{t-i}+\overset{d_{max}}{\underset{j=k+1}{\sum }}\varphi_{1j}Y_{t-j}\\ \;+\overset{k}{\underset{i=1}{\sum }}\delta_{1i}{BL_{vol}}_{t-i}+\overset{d_{max}}{\underset{j=k+1}{\sum }}\delta_{2j}{BL_{vol}}_{t-j}+\overset{k}{\underset{i=1}{\sum }}\theta_{1i}GS_{t-i}\\ \;+\overset{d_{max}}{\underset{j=k+1}{\sum }}\theta_{2j}GS_{t-j}+\varepsilon_{1t} \end{array}$$

(17) $$\begin{array}{l} {FI^{3}}_{t}=\alpha_{0}+\overset{k}{\underset{i=1}{\sum }}\beta_{1i}{FI^{3}}_{t-i}+\overset{d_{max}}{\underset{j=k+1}{\sum }}\beta_{2j}{FI^{3}}_{t-j}+\overset{k}{\underset{i=1}{\sum }}\gamma_{1i}EPU_{t-i}\\ \;+\overset{d_{max}}{\underset{j=k+1}{\sum }}\gamma_{1j}EPU_{t-j}+\overset{k}{\underset{i=1}{\sum }}\varphi_{1i}Y_{t-i}+\overset{d_{max}}{\underset{j=k+1}{\sum }}\varphi_{1j}Y_{t-j}\\ \;+\overset{k}{\underset{i=1}{\sum }}\delta_{1i}{BL_{vol}}_{t-i}+\overset{d_{max}}{\underset{j=k+1}{\sum }}\delta_{2j}{BL_{vol}}_{t-j}+\overset{k}{\underset{i=1}{\sum }}\theta_{1i}GS_{t-i}\\ \;+\overset{d_{max}}{\underset{j=k+1}{\sum }}\theta_{2j}GS_{t-j}+\varepsilon_{1t} \end{array}$$

## Model Estimation and Interpretation

### Unit Root Test

The results of the conventional unit root test, i.e., ADF and P-P *with the null hypothesis of data are not stationary and KPSS with the null hypothesis of data are stationary*, are exhibited in Table 1. Results established a mixed order of integration, suggesting that few variables are stationary at a level *I*(0) and few become stationary after first difference *I*(1). This verdict is pertinent to all three unit root tests.

Table 2 reports the non-linear unit root test results following the study by Kapetanios et al. (2003). The test utilizes three cases, such as raw data (Case 1), the demeaned data (Case 2), and the de-trended data (Case 3), for the series of financial innovation, EPU, gross saving, non-performing loan, and economic growth. This study exposes that the null hypothesis of the *linear unit root test* is rejected for all the variables in either case. Hence, we concluded that the series of financial innovation, EPU, gross savings, non-performing loans, and economic growth follow non-linear stationary processes.

Table 3 displays the results of the non-linear unit root test performed by Kruse (2011). The results signpost that the null hypothesis of the linear unit root test is rejected at either a 1 or 5% level of significance, implying that the series of financial innovation, EPU, gross saving, bad loan, and economic growth follow non-linear stationary processes.

### Non-linearity Test

In the following section, this study investigates both long-run and short-run relationships between financial innovation, EPU, gross savings, non-performing loans, and economic growth of BRIC nations by employing Equation (9). Table 4 displays the results of the ARDL estimation, including the long-run cointegration test in Panel A; long-run coefficients in Panel B; short-run coefficients reports in Panel C; and residual diagnostic test results in Panel D.

**Table 4:** Linear ARDL estimation results.

	**Brazil**			**Russia**			**India**			**China**		
	**[1]**	**[2]**	**[3]**	**[4]**	**[5]**	**[6]**	**[7]**	**[8]**	**[9]**	**[10]**	**[11]**	**[12]**
**Panel A: Long-run cointegration**												
F*_*pss*_*	6.482a	15.623a	7.677a	19.771a	4.79a	5.362a	8.44a	12.102a	12.518a	11.919a	16.761a	9.511a
W*_*pss*_*	12.331a	12.98a	9.609a	10.211a	19.951a	8.96a	19.8a	19.774a	5.101a	11.109a	12.857a	18.106a
t_BDM_	−11.397a	−10.824a	−5.562a	−7.03a	−8.234a	−11.584a	−9.077a	−5.233a	−4.712b	−8.698a	−7.402a	−9.061a
**Panel B: long–run coefficients**												
β	−0.029_a_	−0.026_a_	−0.069_a_	−0.081b	−0.028a	−0.111a	−0.073a	−0.064a	−0.012	−0.074a	−0.053a	−0.029
γ	−0.158_a_	−0.305_a_	0.273_a_	−0.285b	0.182a	−0.331a	0.067b	0.204b	−0.029	−0.019a	−0.115a	0.027a
δ	0.369_a_	0.145_b_	0.549_a_	0.103b	0.089c	0.611c	0.135a	0.681a	0.005	0.045a	0.066a	0.016b
λ	0.172_a_	0.166_b_	0.152_b_	0.154b	0.241b	0.397a	0.139a	0.274a	0.034	0.012a	0.191a	−0.006
**Panel C: Short–run coefficients**												
Constant	−0.341c	0.935c	1.145a	−0.096a	0.098	−0.221	−0.495a	−1.037	−0.015	0.604a	0.201	−0.042
Trend	0.025a	0.104b	−0.04a	0.025a	−0.035	0.013	0.034a	0.019	−0.016	0.061a	0.016	0.242
λ1	−0.034a	0.047b	0.179a	−0.023a	−0.091a	0.123c	−0.325a	−0.291	0.015c	−0.285a	−0.288c	0.073c
λ2	0.016	−0.026b	0.079	0.462	0.913	−0.25	0.021	−0.314	−0.016	0.215a	−0.003	−0.015
λ3	0.038b	0.142a	0.029b	0.145a	0.126a	−0.004	0.015b	0.053c	0.003a	0.081a	0.002	0.006
λ4	−0.014a	−0.014	0.054b	0.213a	−0.196	−0.021	0.001	0.014	−0.011	0.012a	−0.014	−0.004
ζ	−0.104a	−0.084a	−0.091a	−0.123a	−0.081	0.416	−0.317c	−0.314	−0.091	−0.143a	−0.378	−0.053
**Panel D: residual diagnostic test**												
*R*^2^	0.583	0.618	0.146	0.504	0.153	0.439	0.789	0.792	0.329	0.287	0.132	0.747
*F*-test	11.251a	25.315a	0.14.884a	25.015a	75.024a	18.254a	10.384a	10.667a	45.054a	12.587a	15.294a	14.035a
$x_{sRcorr}^{2}$	0.729	0.83	0.446	0.558	0.748	0.66	0.011	0.557	0.271	0.31	0.785	0.237
$x_{Nor}^{2}$	0.543	0.877	0.22	0.41	0.117	0.305	0.368	0.424	0.894	0.942	0.409	0.174
$x_{.hete}^{2}$	0.53	0.749	0.331	0.353	0.751	0.155	0.235	0.078	0.473	0.891	0.714	0.445
RESET	0.918	0.13	0.593	0.321	0.58	0.966	0.584	0.877	0.285	0.152	0.135	0.123

Panel A in Table 4 reports the results of the long-run cointegration test performing three statistics. First, the modified *F*-test (FPSS), introduced by Pesaran et al. (2001); second, a WPSS, which is the above joint null hypothesis; and third, a *t*-test (tBDM) proposed by Banerjee et al. (1998). This study divulges that the null hypothesis of no-cointegration is rejected at a 1% level of significance, suggesting that the test statistics of FPSS, WPSS, and tBDM are higher than the critical value at a 1% level significance. Hence, it is evident that, in the long run, variables in the empirical equation move together. Once the long-run association is documented, this study moves to the next step of figuring out the magnitudes running from EPU to financial innovation both in the long run and short run.

Panel B in Table 4 reports long-run coefficients and reveals adverse effects running from EPU to financial innovation. Results are displayed in column [1] for Brazil, a coefficient of −0.029, [4] for Russia, a coefficient of −0.081, [7] for India, a coefficient of −0.073, and [10] for China, a coefficient of −0.074, where financial innovation has been measured by M2/M1 in the empirical equation. Furthermore, financial innovation proxy by N3/M1 and empirical results are displayed in column [2], a coefficient of −0.026 for Brazil, [5] a coefficient of −0.028 for Russia, [8] a coefficient of −0.064 for India, and [11] a coefficient of −0.053 for China. Furthermore, the empirical model outcome with investment in R&D as a proxy for financial innovation is exhibited in column [3] for Brazil, a coefficient of −0.069, [6] for Russia, a coefficient of 0.073, [9] for India, a coefficient of −0.012, and [12] for China, a coefficient of −0.029. The noticeable fact is that all the coefficients are statistically significant at a 1% level of significance.

Panel C of Table 4 reports the short-run coefficients of the empirical model. This study documents that the error correction terms are negative and statistically significant at a 1% level of significance. This coefficient specifies the speed of adjustments toward long-run equilibrium due to prior period shocks. Regarding the effects of EPU on financial innovation, this study reveals a similar association in a long run, i.e., adverse impact. More precisely, financial innovation proxy by M2/M1 exposes a coefficient of −0.034 for Brazil, a coefficient of −0.023 for Russia, a coefficient of −0.325 for India, and a coefficient of −0.285 for China. Based on the coefficient elasticity, the financial systems of India and China are more responsive than other selected nations.

On the other hand, columns [2], [5], and [8] display the magnitudes of EPU on financial innovation, which are measured by M3/M1. Due to a 10% increase in EPU, the results decline with the speed of financial innovation embellishment by 0.475% in Brazil, by 0.91% in Russia, by 2.91% in India, and by 2.88% in China. The findings suggest that financial innovation in the form of M3/M1 has a more prompt response in India and China due to the movement in EPU. So, it appears that reducing EPU by implementing control mechanisms in the economy, both in India and China, can maximize the potential benefits of Brazil and Russia.

Columns [3], [6], [9], and [12] of Panel C in Table 4 exhibit EPU effects on financial innovation, which are measured by investment in R&D by the financial institution. This study exposes a positive effect running from EPU to financial innovation, i.e., a coefficient of 0.179 for Brazil, a coefficient of 0.123 for Russia, a coefficient of 0.015 for India, and a coefficient of 0.073 for China. These findings suggest that EPU induces financial institutions for expanding their investment in innovating and developing financial services and products to mitigate the adverse effects. Nonetheless, R&D expenditure assists financial institutions in grabbing investment opportunities and reallocating of economic resources in an efficient manner.

For control variables, in the long run, this study discloses that the coefficients of non-performing loans exhibit adverse influence on financial innovation, while gross savings and economic growth appear as motivating factors for the adaptation and evolution of innovative financial products and services in the financial system. Furthermore, the short-run model documents that gross saving plays a positive role in the further development of financial innovation. While non-performing loans and economic growth exhibit adverse influence on financial innovation, their elasticity to financial innovation is statistically insignificant.

Panel D in Table 4 presents the results of diagnostic tests. The associated *p*-value of test statistics is statistically insignificant, implying that empirical models are free from serial correlation, residuals are normally distributed, and internal consistency is also established.

Next, the asymmetric effects of EPU on financial innovation are investigated by executing non-linear ARDL (see Equation 13) and results are reported in Table 5.

**Table 5:** Results of asymmetric model estimation.

**9**	**Brazil**			**Russia**			**India**			**China**		
	**[1]**	**[2]**	**[3]**	**[4]**	**[5]**	**[6]**	**[7]**	**[8]**	**[9]**	**[10]**	**[11]**	**[12]**
**Panel –A: Long–run cointegration test**												
F*_*pss*_*	13.898	14.822	7.544	9.74	9.656	9.999	18.748	5.525	8.606	10.873	12.676	17.409
W*_*pss*_*	13.813	7.353	17.515	7.468	11.08	17.714	16.338	8.303	17.592	10.283	18.387	5.98
t_BDM_	−7.789	−8.058	−11.435	−8.777	−5.588	−4.155	−7.896	−9.861	−7.583	−10.861	−8.378	−12.277
${\text{W}}_{LR}^{\text{EPU}}$	17.668	16.669	8.279	16.006	11.791	18.535	18.961	8.864	8.978	14.461	11.557	19.809
${\text{W}}_{SR}^{\text{EPU}}$	17.668	16.669	8.279	16.006	11.791	18.535	18.961	8.864	8.978	14.461	11.557	19.809
**Panel –B: Long–run coefficients**												
γ^+^	−0.132_a_	−0.041_a_	−0.033_a_	−0.102_a_	−0.312_a_	−0.196_a_	−0.195_b_	−0.004_a_	−0.108_a_	−0.013_a_	−0.101_a_	−0.041_b_
γ^−^	−0.023_a_	−0.025_a_	−0.041_a_	−0.111_a_	−0.051_b_	−0.059_a_	−0.356_a_	−0.015_a_	−0.045_a_	−0.052_b_	−0.107_a_	−0.028_a_
λ	0.348a	0.149b	0.038b	0.091b	−0.486b	−0.27b	0.069b	0.002b	0.195a	−0.106c	−0.031c	0.014c
β	0.174a	0.075b	−0.433b	−0.015b	0.232b	0.114a	−0.127a	0.089b	1.807b	0.135b	0.048a	−0.013c
μ	−0.086b	0.187b	0.031a	0.023a	−0.316a	−0.149	0.159a	−0.17b	−0.481	−0.215a	−0.035c	−0.008
**Panel –C: Short–run coefficients**												
Constant	−0.07	−0.02	7.897	0.464	0.671	−0.251	0.969	0.037	0.943	−0.358	0.204	0.13
Trend	0.006	0.073	−0.024	0.013	−0.002	0.002	−0.081	0.005	−0.024	0.032	0.048	0
δ^+^	−0.016a	0.031*a*	0.371	0.011	−0.012*a*	−0.041b	0.012c	−0.292c	−0.033a	−0.023a	−0.024a	0.209a
δ^−^	0.029	0.078a	0.107	−0.035a	−0.051a	−0.262a	0.098c	0.024c	−0.082a	0.635	−0.011a	0.014
λ	0.043	0.933	0.011	−0.038	0.046	−0.26	0.067	−0.041	−0.864	0.149	−0.015	−0.015
β	0.022	0.125	0.03	0.292	−0.053	0.127	−0.098	−0.003	0.072	−0.119	0.028	0.005
μ	−0.011	−0.193	−0.029	0.058	0.22	0.086	−0.015	0.005	−0.002	0.182	−0.011	−0.004
ζ	−0.123a	−0.24a	−0.373a	−0.306a	−0.194a	−0.133a	−0.262a	−0.094a	−0.328a	−0.128a	−0.091a	−0.083a
**Panel –D: Diagnostic test**												
${\text{W}}_{LR}^{\text{EPU}}$	17.668	16.669	8.279	16.006	11.791	18.535	18.961	8.864	8.978	14.461	11.557	19.809
${\text{W}}_{SR}^{\text{EPU}}$	9.504	5.229	9.616	18.987	6.52	14.332	9.77	4.377	3.502	12.241	8.216	10.728
$x_{Auto}^{2}$	0.343	0.582	0.187	0.063	0.715	0.148	0.699	0.215	0.589	0.494	0.906	0.059
*x*^2^_H**et**_	0.511	0.259	0.168	0.401	0.072	0.29	0.401	0.347	0.907	0.023	0.718	0.535
*x*^2^_Nor_	0.431	0.691	0.866	0.233	0.978	0.342	0.914	0.525	0.212	0.764	0.072	0.859
*x*_RESET_	0.057	0.534	0.168	0.572	0.615	0.493	0.316	0.658	0.427	0.285	0.177	0.177

Panel A in Table 5 displays the results of FPSS, WPSS, and tBDM for the long-run asymmetric cointegration test. The null hypothesis of the symmetry cointegration test is rejected at a 1% level of significance since all the values of test statistics are higher than the critical value. Therefore, the study findings establish the presence of asymmetric cointegration between EPU and financial innovation.

Furthermore, the results of the Wald test reveal the rejection of the null hypothesis, i.e., symmetry in the long run and short run, at a 1% level of significance. These findings suggest that the positive and negative shocks in EPU do not produce linear effects on financial innovation. Therefore, applying NARDL in assessing the long-run and short-run effects of EPU on financial innovation allows a better fit model in empirical estimation.

The non-linear effects of EPU, i.e., positive and negative shocks of EPU, on financial innovation are assessed, and the results for the long run are exhibited in Panel B in Table 5. This study establishes a negative linkage between positive and negative shocks in EPU and financial innovation. These findings suggest that the increase of EPU in the economy has adverse effects on the development and evolution of financial innovation in the financial system; on the other hand, financial stability through reducing EPU acts as a catalyst and encourages financial institutions to adapt and offer innovative financial products and services in the economy. Likewise, the short-run non-linear effects are displayed in Panel C in Table 5. This study reveals several statistically insignificant coefficients. However, we observed that the statistically significant positive and negative shocks established a negative linkage with financial innovation. These findings suggest that EPU can halt the smooth process of financial innovation in the financial system in the short run as policy uncertainty increases financial vitality in the financial system and causes regulatory development.

The results of the long-run and short-run symmetries are exhibited in Panel D in Table 5. Both long-run and short-run asymmetries are evaluated by executing the WPSS with the null hypothesis of “long-run and short-run symmetries.” The test statistics reject the null hypothesis at a 1% level of significance and confirm asymmetry effects running from EPU to financial innovation. These findings postulate that positive and negative shocks in EPU do not occur in the same direction with the same magnitude. Furthermore, the residual diagnostic tests confirm model stability and efficiency for empirical estimation.

Next, this study attempts to gauge the directional causality by employing causal Equations 14–16 and the results of causality are reported in Tables 6–**8**, respectively. This study establishes several directional causalities; however, it focuses on causality between EPU and financial innovation.

**Table 6:** Results of causality test: financial innovation measured by M_2_/M_1_.

	**FI**	**EPU**	**BL**	**GS**	**Y**	**Causal relationship**
**Panel A: for Brazil**						
FI	–	12.761_a_	13.036_a_	6.745_b_	6.268*C*	EPU←→FI; BL←→FI; GS→FI; Y←→FI; BL←→EPU; Y→EPU; EPU→GS; BL→GS; BL→Y
EPU	15.746_a_	–	16.666_a_	2.979	12.19_a_	
BL	7.324_b_	7.608_b_	–	3.554	1.907	
GS	3.839	11.021_a_	11.407_a_	–	0.607	
Y	14.41_a_	3.563	11.453_a_	2.358	–	
**Panel B: for Russia**						
FI	–	8.132_b_	11.388_a_	0.011	1.357	EPU←→FI; BL←→FI;
EPU	9.942_a_	–	0.879	0.975	0.689	
BL	10.463_a_	0.973	–	0.829	3.242	
GS	3.664	2.093	3.437	–	3.755	
Y	2.195	2.476	3.101	0.735	–	
**Panel C: for India**						
FI	–	13.832_a_	4.833	6.317_c_	6.722_c_	EPU←→FI; GS→FI; Y←→FI; BL→EPU; Y←→EPU; FI→BL; Y→BL; EPU→GS; Y←→GS;
EPU	9.075_b_	–	6.837_c_	2.258	12.021_a_	
BL	8.827_b_	0.577	–	0.911	6.924_c_	
GS	1.245	6.995_c_	3.717	–	10.887_a_	
Y	18.231_a_	7.173_c_	5.293	11.606_a_	–	
**Panel D: for China**						
FI	–	12.516_a_	7.764_c_	9.257_b_	4.873	EPU→FI; BL←→FI; GS→FI; BL←→EPU; GS→BL; EPU→Y
EPU	2.145	–	6.569_c_	0.593	2.016	
BL	12.145_a_	6.569_c_	–	8.593_b_	3.016	
GS	1.096	0.97	0.152	–	1.54	
Y	3.899	5.967_c_	0.455	3.53	–	

a,b,c *Specify the level of significance at a 1%, 5%, and 10% respectively*.

**Table 7:** Results of causality test: financial innovation measured by M_3_/M_1_.

	**FI**	**EPU**	**BL**	**GS**	**Y**	**Causal relationship**
**Panel A: Brazil**						
FI		13.595_a_	6.325_c_	3.565	8.304_b_	EPU←→FI; BL←→FI; Y←→FI; BL←→EPU; Y→EPU; GS←→BL; FI→GS; EPU→GS; BL→Y;
EPU	18.823_a_		21.303_a_	3.47	18.845_a_	
BL	9.846_b_	8.293_b_		6.461*c*	1.114	
GS	7.382_c_	10.835_a_	12.549_a_		0.635	
Y	6.779_c_	3.49	12.491_a_	5.028		
**Panel B: Russia**						
FI		6.021_c_	8.353_b_	4.066	3.492	EPU←→FI; BL←→FI; GS→EPU; Y→BL; FI→GS; EPU→Y; GS→Y
EPU	8.047_b_		1.287	11.078_a_	0.815	
BL	15.877_a_	0.383		0.317	7.029_c_	
GS	10.944_a_	2.025	5.012		3.147	
Y	3.989	11.675_a_	5.336	10.497_a_		
**Panel C: India**						
FI		12.142_a_	15.594_a_	7.249_c_	8.072_b_	EPU→FI; BL←→FI; GS→FI; Y←→FI; GS←→EPU; Y←→EPU; Y→BL; Y→GS;
EPU	5.799		7.094_c_	2.026	13.381_a_	
BL	6.119_c_	0.591		1.08	6.114_c_	
GS	0.839	6.733_c_	3.46		9.963_b_	
Y	20.626_a_	6.737_c_	5.689	11.822*a*		
**Panel D: China**						
FI		14.279_a_	10.95_a_	10.225_a_	4.996	EPU←→FI; BL→FI; GS←→FI; BL→EPU; BL→Y
EPU	6.562_c_		7.427_c_	1.446	1.234	
BL	1.422	1.058		0.067	1.486	
GS	1.519	6.392_c_	0.459		0.632	
Y	0.537	1.33	11.852*a*	0.693		

a,b,c *Specify the level of significance at a 1%, 5%, and 10% respectively*.

**Table 8:** Results of causality test: Financial innovation measured by R&D investment by financial institutions.

	**FI**	**EPU**	**BL**	**GS**	**Y**	**Causal relationship**
**Panel A: Brazil**						
FI		6.299_c_	8.494_b_	1.86	0.374	EPU←→FI; BL←→FI; BL←→EPU; Y→EPU; EPU→GS; BL→GS; BL→Y
EPU	12.132_a_		10.335_a_	5.042	8.972_b_	
BL	11.209_a_	8.916_b_		4.741	2.018	
GS	0.765	14.857_a_	14.667_a_		3.504	
Y	0.985	5.968	15.757_a_	4.528		
**Panel B: Russia**						
FI	–	11.113_a_	6.716_c_	11.758_a_	1.026	EPU←→FI; BL→FI; GS→FI; BL→GS; EPU→Y
EPU	11.367_a_	–	1.671	1.757	0.159	
BL	1.442	0.574	–	1.128	4.228	
GS	4.406	1.897	7.066_c_	–	2.277	
Y	1.175	10.698*a*	3.485	0.472	–	
**Panel C: India**						
FI	–	12.858_a_	10.008_a_	2.29	0.864	EPU←→FI; BL→FI; GS→EPU; Y→EPU; GS→BL; FI→GS; BL→Y
EPU	12.618_a_	–	3.774	7.155_c_	13.333_a_	
BL	0.543	4.095	–	7.479_c_	3.524	
GS	11.64_a_	0.945	5.715	1.24	1.31	
Y	1.115	3.009	13.89_a_	4.749	–	
**Panel D: China**						
FI	–	11.999_a_	0.462	10.661_a_	10.257_a_	EPU←→FI; GS→FI; Y→FI; BL→EPU; GS→BL; Y→BL; EPU→GS; FI→Y
EPU	11.883_a_	–	6.556_c_	0.819	4.493	
BL	4.732	2.609	–	8.345_a_	7.649_a_	
GS	0.59	16.029_a_	10.756_a_	–	0.247	
Y	9.934_b_	0.42	0.174	3.788	–	

a,b,c *Specify the level of significance at a 1%, 5%, and 10% respectively*.

Table 6 displays causality results, where financial innovation is measured by the ratio of M2/M1. These study findings divulge that the *feedback hypothesis* holds for explaining the causality between EPU and financial innovation [EPU←→FI] in Brazil, Russia, and India. These findings suggest that shocks, in either case for both variables, are subject to the response. Therefore, the development in financial innovation should be appropriately regulated and evolved in the financial system. Additionally, the unidirectional causal effect is running from EPU to financial innovation [EPU→FI]. Furthermore, the directional association between financial innovation and control variables. This study discloses bidirectional causality between non-performing loans and economic growth, i.e., [BL←→FI; Y←→FI] and unidirectional causality running from gross savings to financial innovation, i.e., [GS→FI].

Table 7 presents causality test results, where M3/M1 measures financial innovation. This study divulges bidirectional causality between EPU and financial innovation [EPU←→FI] in Brazil, Russia, and China. Additionally, unidirectional causality is running from EPU to financial innovation [EPU→FI] in India. Referring to causality between financial innovation and control variables, this study unveils bidirectional causality running between economic growth and financial innovation [Y←→FI] and non-performing loan and financial innovation [BL←→FI] in Brazil, Russia, and India, and gross savings to financial innovation [GS←→ FI] in China. Furthermore, unidirectional causality was revealed to run from gross savings to financial innovation [GS→FI] in India.

The causality results with financial innovation measured by investment in the R&D are reported in Table 8. The study findings support the presence of the *feedback hypothesis* available between EPU and financial innovation [EPU←→FI], i.e., the establishment of bidirectional causality. This verdict applies to all sample countries. Furthermore, the causal effects of the control variable on financial innovation reveal bidirectional causality between non-performing loans and financial innovation [BL←→FI] in Brazil. On the other hand, unidirectional causality runs from non-performing loans to financial innovation [BL→FI] in Russia and India and gross savings to financial innovation [GS→FI] in Russia and China.

## Findings and Policy Implications

The motivation of this study is to unleash fresh evidence for the nexus between financial innovation and EPU in BRIC countries for the period 2004M1–2018M12. To do so, this study applies several econometrical tests, including the non-linear unit root test performed by Kapetanios et al. (2003) and Kruse (2011), non-linearity test through tBDM and non-linear OLS, ARDL performed by Pesaran et al. (2001), and non-linear ARDL introduced by Shin et al. (2014). Furthermore, directional causality is investigated by following the non-Granger causality framework familiarized by Toda and Yamamoto (1995). The key findings of this study are as follows.

First, for detecting the order of integration of variables, both conventional and non-linear unit root tests were utilized. Conventional unit root tests establish a mixed order of integration, i.e., few variables are stationary at a level and others become stationary after first difference. The result of non-linear unit root tests discloses that variables become stationary by following the non-linear process. The presence of a non-linear system in unit root tests induces further estimation following a non-linear framework in the empirical study.

Second, empirical model estimation with ARDL establishes a long-run association between EPU and financial innovation in selected countries. The long-run coefficient exhibits a negative association with different financial innovation proxies, which is obvious in all 12 models. Besides, in the short run, we observed that EPU effects on financial innovation are mostly statistically insignificant. These findings suggest that the control of EPU is unjustifiable for driving financial innovation in the financial system, especially in the long run.

Third, the WPSS statistics confirms that the asymmetric effects run from EPU to financial innovation both in the long run and short run. In the long run, both positive and negative variations in EPU display negative linkage with financial innovation in all empirical models. Considering their elasticity on financial innovation, it appears that negative shocks in EPU are more vibrant than positive shocks in EPU. In the short run, positive and negative shocks in EPU establish a statistically insignificant impact on financial innovation; however, statistically significant coefficients are negatively associated with financial innovation.

Finally, the directional causality test confirms the *feedback hypothesis* for explaining the causal effects between EPU and financial innovation. These findings suggest that in the long run, anything may happen in either variable, i.e., financial innovation or EPU, and the obvious effects will have appeared, respectively. Referring to the causal effect of control variables toward financial innovation, this study establishes unidirectional causality running from control variables to financial innovation in most cases.

With careful consideration of the study findings, we offered the following policy suggestions for future consideration on mitigation of EPU impact on fostering the growth of financial innovation in the economy. First, a state of economic uncertainty has adverse effects on driving the prospects of innovativeness in the financial system and hinders the process of financial development. Thus, it is essential for the policymakers to put considerable efforts into formulating financial policies linked with macro policies for subsidizing the magnitudes for a state of uncertainty. Second, the results of EPU are the accumulated effects of a number of both macro and micro aspect interaction effects. Moreover, strategic policy formulation with respect to monetary and fiscal strategies has to be aligned in such a manner so that the frictions effect in the economy can be minimized as much as possible. Moreover, financial innovation is not only interact with the financial sector but other macro fundamentals those play a pivotal role in the adaptation and diffusion of financial innovation in the economy.

## Data Availability Statement

Publicly available datasets were analyzed in this study. This data can be found at: https://databank.worldbank.org/source/world-development-indicators and https://www.policyuncertainty.com.

## Author Contributions

ZJ: introduction, methodology, and first draft preparation. AM: introduction, empirical model estimation, and final preparation. MQ: introduction, methodology, empirical model estimation, and final preparation. MA: introduction, literature survey methodology, empirical model estimation, and first draft preparation.

## Conflict of Interest

The authors declare that the research was conducted in the absence of any commercial or financial relationships that could be construed as a potential conflict of interest.

## Footnotes

^1^https://www.policyuncertainty.com/
